# Effects of ovarian stimulation protocols on outcomes of assisted reproductive technology in adenomyosis women: a retrospective cohort study

**DOI:** 10.3389/fendo.2023.1198779

**Published:** 2023-08-17

**Authors:** Li Ge, Yexing Li, Shengnan Guan, Linlin Cui, Zi-Jiang Chen

**Affiliations:** ^1^ Center for Reproductive Medicine, the Second Hospital of Shandong University, Cheeloo College of Medicine, Shandong University, Jinan, Shandong, China; ^2^ Center for Reproductive Medicine, Cheeloo College of Medicine, Shandong University, Jinan, Shandong, China; ^3^ Research Unit of Gametogenesis and Health of Assisted Reproductive Technology (ART)-Offspring, Chinese Academy of Medical Sciences (No.2021RU001), Jinan, Shandong, China; ^4^ Key Laboratory of Reproductive Endocrinology of Ministry of Education, Shandong University, Jinan, Shandong, China; ^5^ Shandong Key Laboratory of Reproductive Medicine, Shandong Provincial Hospital Affiliated to Shandong First Medical University, Jinan, China; ^6^ Shandong Provincial Clinical Research Center for Reproductive Health, Jinan, Shandong, China; ^7^ Shandong Technology Innovation Center for Reproductive Health, Jinan, Shandong, China; ^8^ National Research Center for Assisted Reproductive Technology and Reproductive Genetics, Shandong University, Jinan, Shandong, China; ^9^ Shanghai Key Laboratory for Assisted Reproduction and Reproductive Genetics, Shanghai, China; ^10^ Center for Reproductive Medicine, Ren Ji Hospital, School of Medicine, Shanghai Jiao Tong University, Shanghai, China

**Keywords:** adenomyosis, infertility, *in vitro* fertilization (IVF), intracytoplasmic sperm injection (ICSI), pregnancy outcomes

## Abstract

**Objective:**

To evaluate the effects of different ovarian stimulation protocols on in vitro fertilization/intracytoplasmic sperm injection (IVF/ICSI) outcomes in infertile women with adenomyosis.

**Methods:**

We carried out a retrospective cohort study among infertile women with adenomyosis receiving IVF/ICSI treatment, including 257 fresh embryo transfer (ET) cycles and 305 frozen embryo transfer (FET) cycles. In fresh ET cycles, ultra-long, long, short, and antagonist protocols were adopted. In FET cycles, patients received long-acting GnRH agonist (GnRHa) pretreatment or not. The primary outcome was clinical pregnancy rate (CPR), and the secondary outcomes included implantation rate (IR), miscarriage rate (MR), and live birth rate (LBR).

**Results:**

In fresh ET cycles, compared with ultra-long and long protocols, IR (49.7%, 52.1% versus 28.2%, P=0.001) and CPR (64.3%, 57.4% versus 35.6%, P=0.004) significantly decreased in the short protocol. Similarly, compared with ultra-long and long protocols, a decreased inclination of IR (49.7%, 52.1% versus 33.3%) and CPR (57.4%, 64.3% versus 38.2%) existed in the antagonist protocol, although no statistical significance was detected because of strict P adjustment of Bonferroni method (P_adj_=0.008). Compared with long protocol, LBR in short protocol decreased obviously (48.2% versus 20.3%, P<0.001). In FET cycles, no matter which origin of embryos, there were no statistical differences in IR, CPR, and LBR. For women ≥35 years receiving fresh ET, CPR was higher in ultra-long and long protocols (52.1%, 50.0% versus 20.0%, 27.5%, P=0.031) compared to antagonist and short protocols. For women ≥35 years receiving FET, compared with ultra-long and antagonist protocols, cycles with embryos originating from long and short protocols had higher proportions of long-acting GnRHa pretreatment (30.4%,30.00 versus 63.9%, 51.4%, P=0.009). IR (61.1%, 48.6% versus 32.6%, 25.0%, P=0.020) and CPR (58.3%, 48.6% versus 30.4%, 25.0%, P=0.024) in long and short protocols were higher than rates of ultra-long and antagonist protocols, but no statistical differences were supported because of strict Bonferroni method (P_adj_=0.008).

**Conclusion:**

In infertile women with adenomyosis, if a fresh embryo was planned for transfer, an ultra-long or long protocol might be beneficial. If antagonist and short protocols were used, whole embryos frozen followed by FET was recommended. In FET cycles, embryos derived from different protocols had no impact on pregnancy outcomes.

## Introduction

As a continuing conundrum, adenomyosis has besieged clinicians for more than one hundred years, which is manifested in the displacement of endometrial glands and stroma in the myometrium (1). Adenomyosis can induce a series of clinical problems, such as heavy menstrual bleeding, chronic pelvic pain, and infertility (2–4). As time passes, the lesions gradually exacerbate, and eventually result in infertility or other severe impacts. A cross-sectional study showed that the incidence of adenomyosis was 20%-29.7% in the infertile population (5). In infertile women receiving assisted reproductive technology (ART), the proportion could rise to 30%-40% (6).

The negative effect of adenomyosis on ART outcomes was accumulated, and persistent endeavors were made to improve the pregnancy outcomes (6–8). So far, the usage of ultra-long protocol was more extensive because of possible improvement in clinical pregnancy rate (CPR) or live birth rate (LBR) (9–12). A widely accepted mechanism was that downregulation induced by long-acting GnRHa could counter hyperestrogenism and progesterone resistance of adeomyosis (13–15). However, the ultra-long protocol had an obvious defect, that was, deep inhibition of ovarian function, which usually resulted in the increase of the duration and dosage of gonadotropin (10). More seriously, for adenomyosis patients with poorer ovarian reserve, the inhibition of long-acting GnRHa could induce poor ovarian response, manifesting in decreased oocyte retrieval and negative pregnancy outcomes. Besides ultra-long protocol, conventional protocols, such as long, antagonist, and short protocols, all could be adopted, however, systematic evaluation of these protocols was absent. In frozen embryo transfer (FET) cycles, if embryos originated from different protocols, did any differences in pregnancy outcomes exist? There were no answers.

So, we designed this study and tried to systematically evaluate the pregnancy outcomes of different protocols in fresh embryo transfer (ET) cycles. Additionally, we also aimed to elucidate whether embryo deriving from different protocols could affect the outcomes of FET cycles.

## Materials and methods

### Study design and population

Patients with adenomyosis who underwent *in vitro* fertilization/intracytoplasmic sperm injection (IVF/ICSI) at the Center for Reproductive Medicine, Shandong University from January 2016 to December 2020 were included in the retrospective cohort study. The follow-up time was up to April 2023. The study has been reviewed and approved by Ethics Committee at the Center for Reproductive Medicine, Shandong University (No. 2021-133). The inclusion criteria were as follows: (i) age below 42 years at the start of the IVF/ICSI cycles; (ii) diagnosis of adenomyosis based on a consensus opinion from the Morphological Uterus Sonographic Assessment (MUSA) group with subjective enlargement of the uterine corpus and heterogeneous myometrium, accompanying with or without asymmetrical thickening, cysts, hyperechoic islands, fan-shaped shadowing, echogenic subendometrial lines and buds, translesional vascularity, irregular junctional zone and interrupted junctional zone or not (16, 17); (iii) no history of uterine malformation, untreated hydrosalpinx or intrauterine lesions. All scans were performed by experienced imaging doctors, who had over 8 years of experience in gynecological practice. The baseline data of scans were recorded on the electronic medical system.

### Controlled ovarian stimulation protocols during IVF/ICSI

#### Pretreatment

Long acting GnRHa downregulation was an important pretreatment method with duration varing from 1 to 6 cycles according to the response of patients to GnRHa, which was explained in detail in the ultra-long protocol. Surgery and anti-inflammatory drugs were not used as pretreatment.

#### Ultra-long protocol

to receive the first injection of long-acting GnRHa (Triptorelin Acetate®; Ipsen, France; Leuprorelin Acetate®; Lizhu, China) with a dose of 3.75 mg on day 2 or 3 of the menstrual cycle. The anteroposterior diameter of the uterus was measured 28 days after each injection. If the diameter was more than 70 mm, another injection was repeated until the sixth injection. Twenty-eight days after the last dose, the effect of downregulation was evaluated by transvaginal ultrasonography (TVS) and serum hormone examination. Eligible downregulation was defined as endometrium thickness ≤5mm, serum estradiol ≤ 50pg/mL, LH ≤ 5IU/L, and diameters of follicles <8mm in bilateral ovary without functional cysts.

#### Long protocol

A daily dose of triptorelin acetate (0.05–0.1mg) of GnRHa (triptorelin acetate®; Ipsen, France) for 14 days. If downregulation criteria were achieved, ovarian stimulation started.

#### Short protocol

A daily dose (0.05–0.1 mg) of GnRHa (triptorelin acetate®; Ipsen, France) was given on days 2-4 of the menstrual cycle until HCG trigger. After 1-2 days of GnRHa, gonadotropin was used for ovarian stimulation and lasted for about 8-12 days.

#### Antagonist protocol

A daily dose (0.25mg) of GnRH-ant (Orgalutran®, MSD, Netherlands) was used on the fifth or sixth day of gonadotropin until the trigger day.

### Ovarian stimulation

Gonadotropin (Gonal F®, Merck Serono, Switzerland; Lishenbao®, Lizhu, China) at 150–300 IU daily was administered for COS according to age, body mass index (BMI), and ovarian reserve. The adjustment of gonadotropin and addition of recombinant LH (Luveris®, Merck Serono, Germany) were decided by follicular development. Routinely, 8000-10000 IU of urinary human chorionic gonadotropin (HCG) (hCG®; Livzon, China) was used intramuscularly for triggering when at least two follicles measured ≥18mm. If a high risk of ovarian hyperstimulation syndrome (OHSS) existed, 2000-4000 IU of HCG combined with 0.2mg GnRHa was administrated. Oocyte retrieval was performed 34–36h later. The choice of IVF or ICSI depended on sperm quality. Cancellation of fresh ET and whole embryo frozen was carried out in the conditions of high risk of OHSS, hydrosalpinx, or unsynchronized endometrium status. All embryos were cultured for 3 or 5 days with two high-quality cleavage-stage embryos on day 3 or one blastocyst on day 5 transferring into the uterus under the guidance of abdominal ultrasound. The high-quality embryos were defined as 2PN-derived embryos with 7-10 cells and scores ≥3 on day 3 or ≥4BC on day 5 (18). Oral dydrogesterone tablet (Duphaston®, Abbott, Netherlands) 10 mg twice daily and vaginal progesterone gel (Crinone gel®, Merck Serono, Switzerland) 90 mg once daily or oral dydrogesterone tablet (Duphaston®, Abbott, Netherlands) 20 mg twice daily and vaginal progesterone soft capsules (Utrogestan®, Besins, Belgium) 200 mg once daily were administered as luteal phase support.

### Protocols of endometrial preparation in FET cycles

GnRHa pretreatment before FET: GnRHa pretreatment was performed in patients with severer adenomyosis before FET for ≥1 month with 3.75 mg GnRHa per month. The uterine anteroposterior diameter was measured 28 days after each injection. If it was more than 70 mm, injection of the same dose of GnRHa was repeated until the sixth injection. Hormone replacement cycles would be administered 4 weeks after the last dose of GnRHa.

Oral estradiol valerate (Progynova®; Bayer, Germany) was administrated in a dose-escalating method, 4mg/day for the first 5 days and subsequently 6mg/day for the second 5 days. According to the assessment of endometrial thickness and serum hormone levels of E_2_, 8mg/day for another 3-4 days was continued or not. When endometrial thickness ≥7 mm, progesterone addition was started. One frozen-thawed blastocyst was transferred into the uterus 5 days after progesterone addition. Other protocols for endometrial preparation in FET cycles had been described in previous studies (19).

### Observational parameters and outcome variables

Baseline parameters included age, BMI, duration of infertility, primary infertility, antral follicle count (AFC), basal follicle-stimulating hormone (FSH), anti-Müllerian hormone (AMH), the mean diameter of the initial uterus, history of dysmenorrhea, and IVF-related parameters. The mean diameter of the uterus was calculated as the average of long and wide diameter in the longitudinal section measured by TVS on days 2-6 of the menstrual cycle. According to the verbal multidimensional scoring system (20), dysmenorrhea was classified into none, mild, moderate, and severe. The primary pregnancy outcome was CPR, and the secondary pregnancy outcomes included implantation rate (IR), biochemical pregnancy rate (BPR), ectopic pregnancy rate (EPR), miscarriage rate (MR), early MR, late MR, and LBR, cumulative live birth rate (CLBR). Serum β-hCG level was examined 14 days after ET. Biochemical pregnancy as defined as elevated serum β-hCG level ≥10mIU/mL. Clinical pregnancy was defined as the presence of intra-uterine pregnancy or visible extra-uterine pregnancy. Ectopic pregnancy was defined as the presence of a gestational sac or mass outside the uterine cavity. Miscarriage was defined as pregnancy loss before 28 gestational weeks. Clinical pregnancy loss ≤12 gestational weeks was an early miscarriage, vice versa, pregnancy loss >12 gestational weeks was a late miscarriage. IR was defined as the ratio of the total number of gestational sacs confirmed by TVS to the total number of transferred embryos. CLBR was defined as the number of cycles in which patients had the first live birth/number of all transfer cycles per oocyte retrieval, including all fresh and frozen ET.

### Statistical analysis

All statistical analysis was conducted by SPSS 25.0 (SPSS, Chicago, USA). The normality of continuous variables was assessed by the Shapiro-Wilk test. If conforming to normality distribution, mean ± SD and analysis of variance (ANOVA) were adopted for variables description and intra-group comparisons. Vice versa, if the non-normality distribution was ascertained, the median (25th-75th percentile) and Kruskal-Wallis test were used for variables description and intragroup comparisons. Frequencies (percentages) were utilized to describe categorical variables. The comparisons of categorical variables were evaluated by χ (2) test or Fisher’s exact test. Among the pairwise comparisons of four subgroups, the Bonferroni method was used to adjust p-values. In order to account for the differences among subgroups, parameters, such as age, BMI, duration of infertility, types of infertility, AFC, AMH, FSH, initial uterine diameter, and dysmenorrhea history, were included in the logistic regression model. P<0.05 was considered as statistical significance.

## Results

A total of 562 cycles were included with 257 cycles of fresh ET and 305 cycles of FET. In fresh ET cycles, cycles of ultra-long, long, antagonist, and short protocols were 108, 56, 34, and 59, respectively. In FET cycles, cycles of FET with embryos originating from ultra-long, long, antagonist, and short protocols were 98, 101, 54, and 52.

### Pregnancy outcomes of adenomyosis in fresh ET cycles

In fresh ET cycles, except for BMI, there were significant differences in age, duration of infertility, primary infertility, AFC, FSH, AMH, mean initial diameter of the uterus, and dysmenorrhea in intragroup comparisons. Compared with ultra-long, long, and antagonist protocols, the short protocol had older age (34.00, 32.50, 33.50 versus 37.00, P<0.001), poorer AMH (1.65, 2.75, 1.32 versus 1.30, P<0.001), and lower AFC (11.00, 13.00, 10.00versus 9.0, P<0.001). The proportion of severe dysmenorrhea in ultra-long protocol was highest compared with long, antagonist, and short protocols (37.96% versus 17.86%, 11.76%, 13.56%, P<0.001). The data was shown in **Table 1**.

**Table 1:** Baseline characteristics of different COS protocols in fresh ET cycles.

	Ultra-long protocol	Long protocol	Antagonist protocol	Short protocol	P value
No. of cycles	108	56	34	59	
Age, years	34.00 (31.00,37.00)^a^	32.50 (30.00,36.00)^b^	33.50 (32.00,41.00)	37.00 (33.00,40.00)^ab^	0.001
BMI, kg/m2	24.40 (22.09,26.84)	23.37 (21.37,26.63)	25.70 (23.02,27.03)	23.95 (21.76,26.26)	0.249
Duration of infertility, years	3.00 (2.00,5.00)	3.00 (2.00,5.00)	3.50 (2.50,5.50)^d^	2.00 (1.00,3.75)^d^	0.007
Primary infertility, n(%)	40 (37.04)^a^	23 (41.07)^b^	9 (26.47)	6 (10.17)^ab^	<0.001
AFC	11.00 (7.00,14.00)^ac^	13.00 (10.00,16.00)^bce^	10.00 (6.00,14.00)^e^	9.00 (5.00,11.00)^ab^	<0.001
Basal FSH, IU/L	6.45 (5.30,7.78)^a^	6.50 (5.88,7.23)	6.76 (5.60,8.97)	7.60 (6.32,8.87)^a^	0.015
AMH, ng/ml	1.65 (1.06,3.03)^ac^	2.75 (1.66,3.90)^bc^	1.32 (0.92,3.37)	1.30 (0.77,1.90)^ab^	<0.001
Mean initial diameter of uterus, cm	6.22 (5.64,7.30)^ac^	5.43 (4.65,6.35)^c^	6.05 (4.81,6.85)	5.45 (4.72,6.03)^a^	<0.001
History of dysmenorrhea					<0.001
None, n(%)	11 (10.19)	19 (33.93)	16 (47.06)	22 (37.29)	
Mild, n(%)	30 (27.78)	19 (33.93)	8 (23.53)	18 (30.51)	
Moderate, n(%)	26 (24.07)	8 (14.29)	6 (17.65)	11 (18.64)	
Severe, n(%)	41 (37.96)^af^	10 (17.86)	4 (11.76)^f^	8 (13.56)^a^	

Data were presented as median (25th-75th percentile) for non-normality distribution variables and frequencies (percentages) for categorical variables.

COS, controlled ovarian stimulation; BMI, body mass index; AFC, antral follicle count; FSH, follicle stimulating hormone; AMH, anti-müllerian hormone; ET, embryo transfer.

^a^ultra-long vs short; ^b^long vs short; ^c^ultra-long vs long; ^d^antagonist vs short; ^e^long vs antagonist; ^f^ultra-long vs antagonist.

COS-related parameters and pregnancy outcomes were shown in **Table 2**. Compared with the long, antagonist, and short protocols, the duration of gonadotropin (9.00, 9.00, 9.00 versus 11.00, P<0.001) and the dosage of gonadotropin (1800.00, 2175.00, 1975.00 versus 2775.00, P<0.001) increased clearly in the ultra-long protocol. Compared with ultra-long, long and antagonist protocols, endometrial thickness on HCG trigger day was thinnest (1.1, 1.1, 1.0versus 0.9, P=0.002), and the number of retrieved oocytes was lowest (7.00, 10.00, 6.00 versus 4.00, P<0.001) in the short protocol. However, no statistical significance existed among intragroup comparisons of ultra-long, long, and antagonist protocols. For high-quality embryos on day 3, long protocol had more good embryos compared with ultra-long, antagonist, and short protocols (4.00 versus 2.00, 2.00, 2.00, P<0.001).

**Table 2:** Outcomes of ultra-long, long, antagonist, and short protocols in fresh ET cycles.

	Ultra-long protocol	Long protocol	Antagonist protocol	Short protocol	P_adjust_
No. of cycles	108	56	34	59	
Total dosage of Gn, IU	2775.00 (2250.00,3993.75)^acf^	1800.00 (1500.00,2325.00)^c^	2175.00 (1656.25,2728.12)^f^	1975.00 (1425.00,2700.00)^a^	<0.001
Duration of Gn stimulation, days	11.00 (10.00,12.25)^acf^	9.00 (9.00,11.00)^c^	9.00 (8.00,10.00)^f^	9.00 (7.50,10.00)^a^	<0.001
LH on HCG trigger day, IU/L	1.04 (0.56,1.59)^acf^	2.30 (1.43,3.05)^bc^	2.51 (1.80,5.54)^df^	5.24 (3.80,7.04)^abd^	<0.001
E_2_ on HCG trigger day, pg/ml	2061.50 (1407.75,2884.25)	2650.50 (1708.00,3468.25)^be^	1653.50 (1081.00,2172.25)^e^	1619.00 (1294.50,2575.50)^b^	<0.001
P on HCG trigger day, ng/ml	0.66 (0.46,0.84)	0.58 (0.44,0.94)	0.46 (0.35,0.80)	0.65 (0.39,0.81)	0.549
Endometrial thickness on HCG trigger day, cm	1.10 (0.90,1.20)^a^	1.10 (0.90,1.25)^b^	1.00 (0.80,1.10)	0.90 (0.80,1.10)^ab^	0.002
No. of oocytes retrieved	7.00 (4.75,12.00)^a^	10.00 (6.00,12.00)^b^	6.00 (3.00,9.75)	4.00 (3.00,8.00)^ab^	<0.001
No. of 2PN zygotes retrieved	5.00 (3.00,7.25)^a^	6.00 (4.00,9.00)^b^	4.00 (2.25,6.50)	3.00 (2.00,5.00)^ab^	<0.001
No. of high-quality embryos retrieved on Day 3	2.00 (1.00,4.00)^c^	4.00 (2.00,6.00)^bc^	2.00 (2.00,4.00)	2.00 (1.00,3.00)^b^	<0.001
Pregnancy outcomes, %(n/N)					
IR	49.7 (85/171)^a^	52.1 (49/94)^b^	33.3 (18/54)	28.2 (24/85)^ab^	0.001
BPR	10.2 (11/108)	10.7 (6/56)	11.8 (4/34)	8.5 (5/59)	0.960
EPR	0.01 (1/108)	–	–	–	–
CPR	57.4 (62/108)^a^	64.3 (36/56)^b^	38.2 (13/34)	35.6 (21/59)^ab^	0.004
MR	30.6 (19/62)	25.0 (9/36)	30.8 (4/13)	42.9 (9/21)	0.575
Early MR	16.1 (10/62)	22.2 (8/36)	23.1 (3/13)	38.1 (8/21)	0.230^g^
Late MR	14.5 (9/62)	2.8 (1/36)	7.7 (1/13)	4.8 (1/21)	0.241^g^
LBR	39.8 (43/108)	48.2 (27/56)^b^	26.5 (9/34)	20.3 (12/59)^b^	0.007

Data were presented as median (25th-75th percentile) for non-normality distribution variables. Gn, gonadotropin; LH, luteinizing hormone; E2, estradiol; P, progesterone; HCG, human

chorionic gonadotropin IR, implantation rate; BPR, biochemical pregnancy rate; EPR, ectopic pregnancy rate; CPR, clinical pregnancy rate; MR: miscarriage rate; LBR, live birth rate.

^a^ultra-long vs short; ^b^long vs short; ^c^ultra-long vs long; ^d^antagonist vs short; ^e^long vs antagonist; ^f^ultra-long vs antagonist; ^g^Fisher’s exact test.

The parameters of pregnancy outcomes included IR, BPR, EPR, CPR, MR, early MR and late MR, LBR, and CLBR. Compared with ultra-long and long protocols, IR (49.7%, 52.1% versus 28.2%, P=0.001) and CPR (64.3%, 57.4% versus 35.6%, P=0.004) in short protocol significantly decreased. Compared with ultra-long and long protocols, the decrease of IR (49.7%, 52.1% versus 33.3%) and CPR (57.4%, 64.3% versus 38.2%) also existed in antagonist protocol, although no statistical significance was detected because of strict P adjustment of Bonferroni method (P_adj_=0.008). Compared with the long protocol, LBR in the short protocol decreased obviously (48.2% versus 20.3%, P<0.001) and LBR in the antagonist protocol was also pessimistic (48.2% versus 26.5%), although no statistical significance. There were no significant differences in intragroup comparisons of BPR, MR, early MR, and late MR.

Considering the differences in baseline parameters among the four protocols, we carried out CPR and LBR-associated multinomial logistics regression analysis, which was shown in **Supplementary Tables 1**, **2**. When CPR was set as the outcome variable, we observed that long protocol was a protective factor compared with short protocol (OR 2.414, 95% CI 1.011-1.144, P=0.047). AFC was also a protective factor (OR 1.073, 95% CI 1.007-1.144, P=0.030), and the main risk factor was age (OR 0.87, 95% CI 0.809-0.936, P<0.001). When LBR was set as the outcome variable, ultra-long (OR 2.47, 95% CI 1.005-6.07, P=0.049) and long protocol (OR 2.786, 95% CI 1.055-7.353, P=0.039) were both protective factors compared with short protocol. Secondary infertility (OR 2.088, 95% CI 1.018-4.283, P=0.045) and AFC (OR 1.069, 95% CI 1.001-1.142, P=0.047) were also protective factors. Age (OR 0.809, 95% CI 0.743-0.882, P<0.001) was still a risk factor.

### Pregnancy outcomes of adenomyosis in FET cycles

In FET cycles, baseline data of frozen embryo transferred was shown in **Table 3**. Compared with ultra-long, long, and antagonist protocols, the short protocol had older age (33.00, 31.00, 33.00 versus 37.00, P<0.001) and lower AMH (2.84, 4.17, and 4.09 versus 1.35, P<0.001). Age, BMI, duration of infertility, primary infertility, AFC, FSH, and AMH had no statistical difference among ultra-long, long and antagonist protocols. The related parameters of frozen-embryo-originating COS protocols and pregnancy outcomes were shown in **Table 4**. Compared with the long, antagonist, and short protocols, the dosage of gonadotropin (2025.00, 1875.00, and 2025.00 versus 2512.50, P<0.001) and the stimulation duration of gonadotropin (10.00, 9.00, 9.00 versus 11.00, P<0.001) significantly increased in the ultra-long protocol. Ultra-long and long protocols had higher E2 levels on HCG trigger day compared with the short protocol (3117.00, 3000.00 versus 2131.50, P<0.001). The number of retrieved oocytes (6.5 versus 12.00, 12.00, 11.00, P<0.001) and 2PN zygotes (4.00 versus 7.00, 8.00, 6.50, P<0.001) in short protocol were significantly lower than those in ultra-long, long and antagonist protocols. Absolutely different from fresh ET cycles, no statistical differences were detected among ultra-long, long, antagonist and short protocols on IR (45.5%, 47.2%, 41.8%, 50.0%, P=0.851), BPR (12.2%, 6.9%, 5.6%,13.5%, P=0.313), CPR (43.9%, 49.5%, 40.7%, 50.0%, P=0.658), MR (41.9%, 32.0%, 45.5%, 38.5%, P=0.791), early MR (27.9%, 32.0%, 36.4%, 30.8%, P=0.917), late MR (14.0, -, 9.1%, 7.7%, P=0.12), and LBR (25.5%, 33.7%, 22.2%, 30.8%, P=0.403). The CLBR in the long protocol was significantly higher than the corresponding rate in ultra-long, antagonist, and short protocols (68.2% versus 46.2%, 36.2%, 35.9%, p<0.001). We also noticed that in the ultra-long protocol, even if women had severer dysmenorrhea and larger uterus, the CLBR was higher than those in antagonist and short protocol (46.2% versus 36.2%, 35.9%), although no statistical differences existed because of strict P adjustment of Bonferroni method (P_adj_=0.008). So far, 117 blastocytes were still frozen, and we calculated the highest rate of CLBR by hypothesizing that live birth can be achieved by surplus embryo transfer. The real CLBR will fluctuate between the current data and the hypothesized data.

**Table 3:** Baseline characteristics of frozen embryo originating COS cycles.

	Ultra-long protocol	Long protocol	Antagonist protocol	Short group	P value
No. of cycles	98	101	54	52	
Age, years	33.00 (30.00,37.00)^a^	31.00 (30.00,35.00)^b^	33.00 (30.25,36.00)^d^	37.00 (34.00,40.00)^abd^	<0.001
BMI, kg/m2	23.14 (20.70,26.33)^a^	23.28 (21.13,27.18)	24.69 (23.01,26.17)	25.33 (23.06,26.70)^a^	0.016
Duration of infertility, years	3.00 (2.00,4.50)	3.50 (1.50,4.00)	3.50 (2.50,5.50)	2.50 (2.00,4.00)	0.084
Primary infertility, n(%)	51 (52.04)^a^	50 (49.5)^b^	25 (46.3)^d^	7 (13.46)^abd^	<0.001
AFC	13.50 (10.00,19.75)^a^	15.00 (11.00,20.00)^b^	14.00 (9.00,28.5)^d^	9.00 (5.75,12.00)^abd^	<0.001
Basal FSH, IU/L	6.08 (5.06,7.57)	6.16 (5.61,7.03)^b^	6.31 (5.92,7.71)	6.87 (5.57,8.35)^b^	0.013
AMH, ng/ml	2.84 (1.57,5.90)^a^	4.17 (2.78,5.54)^b^	4.09 (1.73,6.90)^d^	1.35 (0.84,2.17)^abd^	<0.001
Mean diameter of initial uterus, cm	6.25 (5.50,7.30)^c^	5.70 (4.70,6.75)^c^	5.80 (4.76,7.05)	5.80 (4.94,6.61)	0.010
History of dysmenorrhea					<0.001
None, n(%)	15 (15.31)	21 (20.79)	15 (27.78)	15 (28.85)	
Mild, n(%)	21 (21.43)	43 (42.57)	13 (24.07)	17 (32.69)	
Moderate, n(%)	26 (26.53)	13 (12.87)	22 (40.74)	9 (17.31)	
Severe, n(%)	36 (36.73)^f^	24 (23.76)	4 (7.41)^f^	11 (21.15)	
long-acting GnRHa pretreatment before FET					0.004
yes	62(63.26%)^c^	39(38.61%)^c^	27(50.00%)	22(42.31%)	
no	36(36.74%)^c^	62(61.39%)^c^	27(50.00%)	30(57.7%)	

Data were presented as median (25th-75th percentile) for non-normality distribution variables and frequencies (percentages) for categorical variables.

BMI, body mass index; AFC, antral follicle count; FSH, follicle-stimulating hormone; AMH, anti-müllerian hormone; COS, controlled ovarian stimulation; FET, frozen embryo transplant.

^a^ultra-long vs short; ^b^long vs short; ^c^ultra-long vs long; ^d^antagonist vs short; ^e^long vs antagonist; ^f^ultra-long vs antagonist.

**Table 4:** Outcomes of FET and characteristics of frozen embryo originating COS cycles.

	Ultra-long protocol	Long protocol	Antagonist protocol	Short protocol	P_adjust_
No. of cycles	98	101	54	52	
Total dosage of Gn, IU	2512.50 (2025.00,3862.50)^acf^	2025.00 (1500.00,2775.00)^c^	1875.00 (1418.75,2531.25)^f^	2025.00 (1556.25,2625.00)^a^	<0.001
Duration of Gn stimulation, days	11.00 (10.00,12.75)^acf^	10.00 (9.00,12.00)^c^	9.00 (9.00,11.00)^f^	9.00 (8.00,10.25)^a^	<0.001
LH on HCG trigger day, IU/L	1.00 (0.57,1.40)^acf^	1.74 (1.15,2.61)^bce^	3.28 (1.98,4.92)^ef^	4.79 (3.40,6.50)^ab^	<0.001
E_2_ on HCG trigger day, pg/ml	3117.00 (2445.00,4806.75)^a^	3000.00 (2373.00,4300.00)^b^	2158.00 (1741.00,4566.25)	2131.50 (1597.75,3408.25)^ab^	<0.001
P on HCG trigger day, ng/ml	0.75 (0.53,0.99)	0.74 (0.50,1.08)	0.83 (0.43,1.18)	0.71 (0.43,0.91)	0.542
Endometrial thickness on HCG trigger day, cm	0.90 (0.80,1.00)	0.90 (0.80,1.00)	0.90 (0.80,1.00)	0.88 (0.80,1.00)	0.676
No. of oocytes retrieved	12.00 (9.00,17.75)^a^	12.00 (10.00,16.00)^b^	11.00 (5.25,15.00)^d^	6.50 (4.00,8.25)^abd^	<0.001
No. of 2PN zygotes retrieved	7.00 (5.00,12.00)^a^	8.00 (6.00,10.00)^b^	6.50 (4.00,10.00)^d^	4.00 (3.00,6.00)^abd^	<0.001
No. of high-quality embryos retrieved on Day 3	4.00 (2.00,6.00)	5.00 (3.00,8.00)^b^	3.00 (1.25,6.00)	3.00 (2.00,4.00)^b^	<0.001
Pregnancy outcomes, %(n/N)					
IR	45.5 (45/99)	47.2 (51/108)	41.8 (23/55)	50.0 (26/52)	0.851
BPR	56.1(55/98)	56.4 (57/101)	46.3 (25/54)	63.5 (33/52)	0.313
EPR	–	–	–	0.02 (1/52)	–
CPR	43.9 (43/98)	49.5 (50/101)	40.7 (22/54)	50.0 (26/52)	0.658
MR	41.9 (18/43)	32.0 (16/50)	45.5 (10/22)	38.5 (10/26)	0.791
Early MR	27.9 (12/43)	32.0 (16/50)	36.4 (8/22)	30.8 (8/26)	0.917
Late MR	14.0 (6/43)	–	9.1 (2/22)	7.7 (2/26)	0.120^g^
LBR	25.5 (25/98)	33.7 (34/101)	22.2 (12/54)	30.8 (16/52)	0.403
CLBR	46.2(67/145)^c^	68.2(60/88)^bce^	36.2(21/58)^e^	35.9(21/58)^b^	<0.001
CLBR^hypo^	59.8(116/194)^c^	76.5(91/119)^bce^	50.7(38/75)^e^	49(48/98)^b^	<0.001

Data were presented as median (25th-75th percentile) for non-normality distribution variables. Gn, gonadotropin; LH, luteinizing hormone; E2, estradiol; P, progesterone; HCG, human

chorionic gonadotropin IR, implantation rate; BPR, biochemical pregnancy rate; EPR, ectopic pregnancy rate; CPR, clinical pregnancy rate; MR, miscarriage rate; LBR, live birth rate;

CLBR, cumulative live birth rate, was calculated based on current finished embryo transfer cycles. CLBR^hypo^ was calculated by hypothesizing that all surplus embryos were transferred and

live birth was achieved. ^a^ultra-long vs short; ^b^long vs short; ^c^ultra-long vs long; ^d^antagonist vs short; ^e^long vs antagonist; ^f^ultra-long vs antagonist; ^g^Fisher’s exact test.

### Pregnancy outcomes of adenomyosis with age ≥35 years in fresh ET and FET cycles

Considering the possible selection bias of different protocols, we carried out an analysis among women≥35 years. In fresh ET cycles, cycles of ultra-long, long, antagonist, and short protocols were 48, 20, 15, and 40, respectively. There were no significant differences in BMI, basal FSH, AMH, and mean diameter of the initial uterus (**Supplementary Table 3**).

Antagonist protocol had significantly older age compared with ultra-long, long, and short protocols (42.00 versus 37.00, 38.00, and 39.00, P=0.002). The proportion of no dysmenorrhea in short protocol was significantly higher than in ultra-long protocols (45.0% versus 10.40%, P=0.010). Compared with antagonist and short protocols, ultra-long and long protocols had more number of oocytes (3.00, 4.00 versus 6.00, 8.00, P=0.02, P=0.011) and 2PN zygotes (3.00, 2.00 versus 4.50, 5.00, P=0.012). However, there was no statistical difference in high-quality embryos on day 3. As to the pregnancy outcomes, ultra-long and long protocols had higher CPR (52.1%, 50.00% versus 20.0%, 27.5%, P=0.031). Although there were no significant differences on IR (P=0.183), BPR (P=0.87), MR (P=0.417), and LBR (P=0.071), LBR in ultra-long and long protocols was higher than the corresponding rates in antagonist and short protocols (27.1%, 30.0% versus 6.7%, 10.0%). The data was shown in **Supplementary Table 4**).

In FET cycles, the FET cycles with embryos originating from ultra-long, long, antagonist, and short protocols were 46, 36, 20, and 37, respectively. The data was shown in **Supplementary Tables 5**, **6**. No significant differences existed in age between the four groups (P=0.331). Except for BMI, there were no statistical differences in the duration of infertility, the proportion of primary infertility, basal FSH, AMH, initial mean diameter of the uterus, the duration and dosage of gonadotropin, oocyte retrieved, number of 2PN zygotes and the final number of high-quality embryos retrieved on Day 3. The proportion of long-acting GnRHa pretreatment before FET in the long and short protocols groups was higher than in the ultra-long and antagonist groups (63.9%, 51.4% versus 30.4%, 30.00%, P=0.009). IR (61.1%, 48.6% versus 32.6%, 25.0%, P=0.020) and CPR (58.3%, 48.6% versus 30.4%, 25.0%, P=0.024) in long and short protocols were higher than rates of ultra-long and antagonist protocols, but no statistical differences were supported because of strict Bonferroni method (Padj=0.008). No significant differences in BPR (P=0.338), MR (P=0.634), and LBR (P=0.078) were detected. The CLBR in long protocol was significantly higher than those in ultra-long, antagonist, and short protocols (68.8% versus 34.4%, 16%, 26.4%, P<0.001). In the ultra-long protocol, an even higher proportion of dysmenorrhea and larger uterus existed, and the CLBR was better compared with the antagonist and short protocols (34.4% versus 16%,26.4%), although no statistical differences existed because of strict P adjustment of Bonferroni method (P_adj_=0.008).

We carried out multinomial logistic regression analysis and CPR was set as the outcome variable. In fresh ET cycles, older age was an important risk factor (OR 0.787, 95% CI 0.658-0.941, P=0.008) and no protective factors were observed (**Supplementary Table 7**). In FET cycles, AFC (OR 0.903, 95% CI 0.821-0.994, P=0.037) and secondary infertility (OR 0.200, 95% CI 0.058-0.690, P=0.011) were observed as protective factors in FET cycles, which were shown in **Supplementary Table 8**.

## Discussion

Ultra-long protocol could mediate hypo-estrogen status caused by pituitary downregulation and produce positive effects on pregnancy outcomes (21). The long protocol also could mediate pituitary downregulation with weaker function. Because of endogenous inhibition of GnRHa, the duration and dosage of gonadotropin significantly increased in the ultra-long protocol. There were no statistical differences in the number of retrieved oocytes and 2PN zygotes in the intragroup comparison of ultra-long and long protocols. In our study, the ultra-long protocol had a higher proportion of severe adenomyosis, however, after long-acting GnRHa pretreatment, IR, CPR, MR, and LBR in ultra-long and long protocols were similar. Hou X’s study analyzed 362 ultra-long protocol cycles and 127 long protocol cycles in fresh ET cycles among adenomyosis patients, and the dosage and duration of gonadotropin in ultra-long protocol also significantly increased. Compared with the long protocol, CPR and LBR in the ultra-long protocol were higher (10). Lan J’s study ascertained that the ultra-long protocol improved pregnancy outcomes in women with adenomyosis, especially in women with diffuse adenomyosis (12). All in all, the ultra-long protocol had its advantages for the improvement of pregnancy outcomes for severer adenomyosis, although time and economic cost was increased.

Antagonists could produce a rapid suppression of pituitary function, which could decrease the dosage of gonadotropin and the risk of ovarian hyperstimulation (22). Compared with ultra-long and long protocols, AFC in antagonist protocol was lower, however, the number of retrieved oocytes and 2PN zygotes had no statistical significance. Compare with ultra-long and long protocols, IR, CPR, and LBR in antagonist protocol were poorer, however, strict P adjustment of the Bonferroni method (P_adj_=0.008) in pairwise under multiple comparisons made the statistical difference difficult to achieve. The BPR, MR, early MR, and late MR were similar among ultra-long, long, and antagonist protocols. Thalluri V. reported CPR in antagonist protocol was 23.6% in patients with adenomyosis, which was similar to our data and significantly lower than the reported CPR in ultra-long or long protocols (9–11, 23). Kolanska K compared pregnancy outcomes of GnRH-agonist versus GnRH-antagonist protocols in women with endometriosis-associated infertility and inferred GnRH-antagonist associated dysfunction of endometrial receptivity might result in decreased CPR and LBR (24).

The advantages of a short protocol could promote the secretion of FSH by flare-up effect, strengthen recruitment of early follicles and improve the response of the ovaries. Compared with ultra-long and long protocols, CPR and LBR in the short protocol were significantly decreased. Vice versa, compared with antagonist protocol, there were no statistical differences in IR, CPR, MR, and LBR. Sheng compared pregnancy outcomes of ultra-long, long, and short protocols, and CPR in the short protocol was 22.4% (25), which was similar to our data. Khan KN et al. reported short and antagonist protocols could not induce hypo-estrogenism and recover potential endometrial normalization of adenomyosis (14, 26), so when they were used, fresh ET should be prudent because of the obviously negative pregnancy outcomes.

In FET cycles, except for larger uterine diameter and a higher proportion of severe dysmenorrhea in ultra-long protocols, other baseline data of embryos originating COS cycles were similar in ultra-long, long and antagonist protocols. However, baseline differences between short and other protocols still had statistical differences, such as age, AFC, and AMH. What we should pay attention to is that no matter which was the origin of the embryo, IR, CPR, MR, and LBR had no statistical difference in FET cycles. So far, there were no other similar comparisons. As to CLBR, because patients still had frozen embryos, we calculated fluctuation ranges by hypothesizing successful live birth with embryo transfer in the future. Compared with other protocols, current data showed that long protocol had its advantages on CLBR. The baseline parameters between long and antagonist were matched, however, the CLBR was poor in antagonist protocol, therefore, antagonist protocol should be cautiously adapted. For severer adenomyosis with good ovarian reserve, the ultra-long protocol was worth considering, while more data was needed to verify a better choice between ultra-long and long protocol.

Considering the possible choosing bias, we analyzed the usage of different protocols and pregnancy outcomes among women ≥35 years. In fresh ET cycles, the antagonist protocol had older age than the ultra-long protocol, while no difference in age existed in other intragroup comparisons. AMH and the final number of high-quality embryos had no statistical significance. Compared with the antagonist and short protocols, CPR in ultra-long and long protocols was higher. LBR in antagonist and short protocols were lower, although no statistical difference existed. In FET cycles, embryos from long and short protocols had a higher proportion of long-acting GnRHa pretreatment, which IR and CPR increased, indicating the possible benefit of long-acting GnRHa.

## Strengths and limitations

This was the first study to make direct comparisons of different COS protocols on IVF/ICSI outcomes.Our study indicated that an ultra-long protocol might be a better choice for severer adenomyosis in fresh ET cycles.We observed that the pregnancy outcomes of antagonist and short protocols were poor in fresh ET cycles, however, the outcomes could be reversed by whole embryos being frozen and a FET strategy. The principal limitation of the study was the possible bias of a retrospective cohort study, therefore, we developed strict inclusion and exclusion criteria to minimize the bias. Prospective studies are needed to verify the conclusions.

## Conclusion

If fresh ET was decided upon, an ultra-long or long protocol might be appropriate. If antagonist and short protocols were used, whole embryo frozen combed with FET is recommended. In FET cycles, embryo origin had no impact on pregnancy outcomes.

## Data availability statement

Data will be made available to the editors of the journal for review or query upon request.

## Ethics statement

The studies involving humans were approved by Ethics Committee at the Center for Reproductive Medicine, Shandong University (No. 2021-133). The studies were conducted in accordance with the local legislation and institutional requirements. The participants provided their written informed consent to participate in this study.

## Author contributions

LG and YL analyzed data and drafted the article. SG collected and analyzed data. LC and Z-JC designed, revised, and approved the manuscript.
