# Supplementary material for: Effects of ovarian stimulation protocols on outcomes of assisted reproductive technology in adenomyosis women: a retrospective cohort study
Source: Front Endocrinol (Lausanne). 2023 Aug 17;14:1198779. doi: 10.3389/fendo.2023.1198779 (PMC10472936; doi:10.3389/fendo.2023.1198779)
Supplement: Supplementary file 1 [file DataSheet_1.pdf]

Supplementary Table I Multivariate logistic regression of clinical pregnancy in fresh ET cycles

	B	*SE*	*Wald*	*P*	*OR*	*95%CI*
Age	-0.139	0.037	14.04	<0.001	0.87	0.809-0.936
BMI	-0.052	0.04	1.702	0.192	0.949	0.877-1.027
Duration of infertility	0.016	0.049	0.1	0.752	1.016	0.922-1.119
Primary infertility	control					
Secondary infertility	0.452	0.349	1.672	0.196	1.571	0.792-3.115
AFC	0.071	0.033	4.737	0.030	1.073	1.007-1.144
AMH	-0.145	0.091	2.56	0.110	0.865	0.724-1.033
Basal FSH	0.086	0.063	1.893	0.169	1.09	0.964-1.233
initial uterine diameter	0.005	0.123	0.002	0.967	1.005	0.789-1.28
None dysmenorrhea	control					
Mild dysmenorrhea	0.113	0.387	0.086	0.769	1.12	0.525-2.391
Moderate dysmenorrhea	0.206	0.436	0.223	0.637	1.229	0.523-2.888
Severe dysmenorrhea	0.522	0.444	1.379	0.24	1.685	0.705-4.023
COSGroup(ultra-long vs short)	0.661	0.402	2.698	0.1	1.937	0.88-4.261
COSGroup(long vs short)	0.881	0.444	3.934	0.047	2.414	1.011-5.767
COSGroup(antagonist vs short)	0.103	0.507	0.042	0.838	1.109	0.41-2.996
COSGroup(ultra-long vs antagonist)	0.557	0.464	1.443	0.230	1.746	0.703-4.336
COSGroup(long vs antagonist)	0.778	0.494	2.481	0.115	2.177	0.827-5.730
COSGroup(ultra-long vs long)	-0.22	0.393	0.314	0.575	0.802	0.371-1.734

BMI: body mass index; AFC: antral follicle count; FSH: follicle stimulating hormone; AMH: anti-müllerian hormone; COS: controlled ovarian stimulation;

Supplementary Table II Multivariate logistic regression of live birth in fresh ET cycles

	B	*SE*	*Wald*	*P*	*OR*	*95%CI*
Age	-0.211	0.044	23.354	<0.001	0.809	0.743-0.882
BMI	-0.077	0.043	3.185	0.074	0.926	0.85-1.008
Duration of infertility	0.069	0.054	1.681	0.195	1.072	0.965-1.191
Primary infertility	control					
Secondary infertility	0.736	0.367	4.033	0.045	2.088	1.018-4.283
AFC	0.067	0.034	3.928	0.047	1.069	1.001-1.142
AMH	-0.226	0.104	4.751	0.029	0.798	0.651-0.977
Basal FSH	-0.005	0.071	0.005	0.945	0.995	0.866-1.143
initial uterine diameter	-0.049	0.135	0.132	0.716	0.952	0.731-1.24
None dysmenorrhea	control					
Mild dysmenorrhea	-0.346	0.426	0.659	0.417	0.708	0.307-1.631
Moderate dysmenorrhea	-0.177	0.468	0.143	0.706	0.838	0.335-2.096
Severe dysmenorrhea	-0.093	0.468	0.04	0.842	0.911	0.364-2.28
COSGroup(ultra-long vs short)	0.904	0.459	3.888	0.049	2.47	1.005-6.07
COSGroup(long vs short)	1.024	0.495	4.28	0.039	2.786	1.055-7.353
COSGroup(antagonist vs short)	0.253	0.597	0.18	0.672	1.288	0.4-4.148
COSGroup(ultra-long vs antagonist)	0.651	0.527	1.528	0.216	1.918	0.683-5.388
COSGroup(long vs antagonist)	0.771	0.537	2.067	0.151	2.163	0.756-6.192
COSGroup(ultra-long vs long)	-0.12	0.397	0.092	0.762	0.887	0.407-1.931

BMI: body mass index; AFC: antral follicle count; FSH: follicle stimulating hormone; AMH: anti-müllerian hormone; COS: controlled ovarian stimulation;

Supplementary Table III Baseline characteristics of women ≥35 years with different COS protocols in fresh ET cycles

	Ultra-long protocol	Long protocol	Antagonist protocol	Short protocol	P value
No. of cycles	48	20	15	40	
Age, years	37.00(36.00,39.00)[f]	38.00 (36.00,41.00)	42.00 (38.50,44.00)[f]	39.00 (37.00,42.00)	0.002
BMI, kg/m2	25.05 (22.29,27.81)	24.22 (20.53,27.91)	25.82(23.57,28.07)	24.94 (21.85,28.03)	0.474
Duration of infertility, years	3.00 (1.08,5.50)	3.25 (2.00,5.00)	3.00 (2.00,7.00)[d]	2.00 (1.00,3.00)[d]	0.009
Primary infertility, n(%)	9 (18.8)[a]	2(10.00)	2(13.3)	0 (0.00)[a]	0.003
AFC	11.00(5.06, 7.83)	13.00 (10.00,16.00)[e]	10.00 (6.00,14.00)[e]	9.00 (5.00,11.00)	0.019
Basal FSH, IU/L	6.10 (5.06,7.82)	6.52 (5.94,7.86)	7.68 (6.55,9.90)	7.09 (6.19,8.90)	0.050
AMH, ng/ml	1.54 (1.01,2.84)	1.76 (1.02,2.67)	1.06 (0.55,3.18)	1.33 (0.76,2.15)	0.285
Mean diameter of initial uterus, cm	6.70 (6.03,8.10)	5.35 (4.79,6.25)	5.35 (4.79,6.25)	5.62(4.76,6.27)	0.100
History of dysmenorrhea					0.010
None, n(%)	5 (10.40)[a]	7(35.00)	5 (33.33)	18(45.00)[a]	
Mild, n(%)	13 (27.10)	7(35.00)	6 (40.00)	12 (30.00)	
Moderate, n(%)	16 (33.30)	3 (15.00)	3 (20.00)	5 (12.50)	
Severe, n(%)	14(29.20)	3 (15.00)	1 (6.70)	5(12.50)	

Data were presented as median (25th-75th percentile) for non-normality distribution variables and frequencies (percentages) for categorical variables.
COS: controlled ovarian stimulation; BMI: body mass index; AFC: antral follicle count; FSH: follicle stimulating hormone; AMH: anti-müllerian hormone; ET embryo transfer
[a] ultra-long vs short; [b] long vs short; [c] ultra-long vs long; [d] antagonist vs short; [e] long vs antagonist; [f] ultra-long vs antagonist

Supplementary Table IV Outcomes of women ≥35 years with different protocols in fresh ET cycles

	Ultra-long protocol	Long protocol	Antagonist protocol	Short protocol	P_{adjust}
No. of cycles	48	20	15	40	
Total dosage of Gn, IU	2850.00 (2343.75, 4031.25)[ac]	1800.00 (1387.50,2325.00)[c]	2400.00 (1675.00,2950.00)	2025.00 (1575.00,2700.00)[a]	<0.001
Duration of Gn stimulation, days	11.00 (9.25,12.75)[acf]	9.00 (8.25,10.00)[c]	9.00 (8.00,10.00)[f]	9.00 (8.00,10.00)[a]	<0.001
LH on HCG trigger day, IU/L	0.98 (0.51,1.66)[acf]	2.45 (1.97,3.34)[bc]	3.50 (2.10,6.29)[df]	5.25 (3.60,7.30)[abd]	<0.001
E_2 on HCG trigger day, pg/ml	1836.50 (1186.00,2575.00)	2374.50 (1495.75, 2994.50)	1230.00 (958.00,1600.00)	1641.00 (1243.75,2483.00)	0.08
P on HCG trigger day, ng/ml	0.63 (0.40,0.83)	0.52 (0.41,0.77)	0.39 (0.34,0.54)	0.57 (0.34,0.75)	0.317
Endometrial thickness on HCG trigger day, cm	1.10 (0.90,1.20)[a]	1.00 (0.91,1.24)[b]	1.00 (0.80,1.10)	0.90 (0.80,1.08)[ab]	0.015
No. of oocytes retrieved	6.00 (4.00,11.75)	8.00 (4.25,10.00)	3.00 (2.00,6.00)	4.00 (3.00,8.00)	0.011
No. of 2PN zygotes retrieved	4.50 (2.25,7.50)[a]	5.00 (3.00,8.75)	3.00 (2.00,5.00)	2.00 (2.00,5.00)[a]	0.012
No. of high-quality embryos retrieved on Day 3	2.00 (1.00,3.75)	3.00 (2.00,4.75)	2.00 (1.00,2.00)	1.00 (1.00,3.00)	0.059
Pregnancy outcomes, %(n/N)					
IR	41.3 (31/75)	36.4 (12/33)	16.7 (4/24)	37.5(12/32)	0.183
BPR	62.5(30/48)	60.0 (12/20)	33.3 (5/15)	35.0 (14/40)	0.87
CPR	52.1 (25/48)	50.0 (10/20)	20.0(3/15)	27.5 (11/40)	0.031
MR	48.0(12/25)	40.0 (4/10)	66.7 (2/3)	63.6 (7/11)	0.417
Early MR	28.0 (7/25)	30.0 (3/10)	66.7(2/3)	54.5 (6/11)	0.288
Late MR	20.0(5/25)	10.0 (1/10)	0.00(0/3)	9.1 (1/11)	0.846
LBR	27.1 (13/48)	30.0 (6/20)	6.7% (1/15)	10.0 (4/40)	0.071

Data were presented as median (25th-75th percentile) for non-normality distribution variables. Gn: gonadotropin; LH: luteinizing hormone; E2: estradiol; P: progesterone; HCG: human chorionic gonadotropin IR: implantation rate; BPR: biochemical pregnancy rate; EPR: ectopic pregnancy rate; CPR: clinical pregnancy rate; MR: miscarriage rate; LBR: live birth rate
[a]ultra-long vs short; [b]long vs short; [c]ultra-long vs long; [d]antagonist vs short; [e]long vs antagonist; [f]ultra-long vs antagonist [g]Fisher's exact test

Supplementary Table V Baseline characteristics of women≥35 years receiving FET with frozen embryo originating COS cycles

	Ultra-long protocol	Long protocol	Antagonist protocol	Short protocol	P
No. of cycles	46	36	20	37	
Age, years	37.00 (36.00,40.00)	37.00 (35.00,41.00)	38.00 (35.25,40.75)	38.00 (36.00,41.00)	0.331
BMI, kg/m2	23.85 (22.69,27.21)[c]	22.14(20.73,24.67)[bce]	25.28 (23.81,26.07)[e]	25.33 (23.36,26.75)[b]	0.001
Duration of infertility, years	3.25 (1.38,7.00)[ac]	2.00 (1.00,4.00)[ce]	3.25 (2.00,7.38)[e]	2.50 (1.50,3.00)[a]	0.038
Primary infertility, n(%)	14 (30.4)[a]	6 (16.7)[b]	4 (20.0)[d]	2 (5.4)[abd]	0.028
AFC	12.00 (9.00,15.25)	13.50 (10.00,18.75)[b]	10.50 (8.25,14.0)	11.00 (6.00,12.00)[b]	0.004
Basal FSH, IU/L	5.98 (5.11,7.25)[af]	6.07 (5.11,7.07)[be]	6.72 (5.74,9.73)[ef]	6.92 (5.57,7.73)[ab]	0.029
AMH, ng/ml	2.16 (1.46,4.69)[a]	2.78 (1.53,4.37)[b]	2.00 (1.10,6.00)[d]	1.36 (0.86,2.17)[abd]	0.002
Mean diameter of initial uterus, cm	7.00 (6.15,7.76)[ac]	5.75 (4.80,6.70)[c]	6.50 (5.09,8.48)	5.85 (4.98,6.48)[a]	<0.001
History of dysmenorrhea					0.03
None, n(%)	9 (19.6)[f]	10 (27.8)	0 (0.00)[df]	12 (32.4)[d]	
Mild, n(%)	13 (28.3)	15(41.7)	8 (40.0)	14 (37.8)	
Moderate, n(%)	10 (21.7)[f]	5 (13.9)[b]	11 (55.0)[fd]	6 (16.2)[bd]	
Severe, n(%)	14 (30.4)	6 (16.7)	1 (5.0)	5 (13.5)	
long acting GnRHa pretreatments before					0.009
yes	14(30.4%)[c]	23(63.9%)[ce]	6(30.00%)[de]	19(51.4%)[d]	
no	32(69.6%)[c]	13(36.1%)[c]	14(70.00%)	18(48.6%)	

Data were presented as median (25th-75th percentile) for non-normality distribution variables and frequencies (percentages) for categorical variables.
BMI: body mass index; AFC: antral follicle count; FSH: follicle stimulating hormone; AMH: anti-müllerian hormone; COS: controlled ovarian stimulation; FET: frozen embryo transplant
[a] ultra-long vs short; [b] long vs short; [c] ultra-long vs long; [d] antagonist vs short; [e] long vs antagonist; [f] ultra-long vs antagonist

Supplementary Table VI Pregnancy outcomes and COS characteristics of women≥35 years receiving FET with frozen embryo originating COS cycles

	Ultra-long protocol	Long protocol	Antagonist protocol	Short protocol	P_{adjust}
No. of cycles	46	36	20	37	
Total dosage of Gn, IU	2512.50 (2081.25,3637.50)[ac]	1987.50 (1350.00,2475.00)[c]	2437.50 (1625.00,3043.75)	2025.00 (1612.50,2625.00)[a]	0.005
Duration of Gn stimulation, days	11.00 (10.00,12.25)[acf]	10.00 (9.00,11.75)[c]	9.00 (9.00,10.75)[f]	9.00 (8.00,10.50)[a]	<0.001
LH on HCG trigger day, IU/L	0.97 (0.62,1.28)[acf]	1.93 (1.31,2.36)[bc]	3.47 (1.84,4.90)[f]	5.25 (3.64,6.71)[ab]	<0.001
E_2 on HCG trigger day, pg/ml	3000.00 (2135.50,4000.00)	2756.00 (2003.00,3746.00)	1957.50 (1142.00,3358.00)	1816.00 (1587.00,3167.50)	0.069
P on HCG trigger day, ng/ml	0.65 (0.46,0.93)	0.65 (0.46,0.83)	0.44 (0.27,1.19)	0.73 (0.43,0.91)	0.900
Endometrial thickness on HCG trigger day, cm	0.93 (0.75,1.00)	0.90 (0.81,1.08)	0.90 (0.80,1.08)	0.85 (0.73,1.00)	0.623
No. of oocytes retrieved	11.50 (9.00,15.25)[af]	10.00 (7.5,15.75)[b]	6.00 (4.00,14.00)[f]	5.00 (4.00,9.00)[ab]	<0.001
No. of 2PN zygotes retrieved	7.00 (5.00,12.00)[a]	8.00 (5.00,9.00)[b]	4.00 (3.00,9.50)	4.00 (3.00,6.50)[ab]	<0.001
No. of high-quality embryos retrieved on Day 3	3.00 (2.00,6.25)	4.00 (2.00,5.00)	2.5 (1.00,4.00)	3.00 (2.00,4.00)	0.104
Pregnancy outcomes, %(n/N)					
IR	32.6 (15/46)	61.1 (22/36)	25.0 (5/20)	48.6 (18/37)	0.020
BPR	47.8(22/46)	63.9 (23/38)	30.0 (6/20)	56.7(21/37)	0.338
CPR	30.4 (14/46)	58.3 (21/36)	25.0(5/20)	48.6 (18/37)	0.024
MR	35.7 (5/14)	23.8(5/21)	40.0(2/5)	44.4(8/18)	0.634
Early MR	28.6 (4/14)	23.8 (5/21)	40.0 (2/5)	38.9 (7/18)	0.732
Late MR	7.1 (1/14)	-	-	5.6 (1/18)	0.599
LBR	19.6 (9/46)	42.9 (15/35)	15.0 (3/20)	27.0 (10/37)	0.078
CLBR	34.4(22/64)[c]	68.8(22/32)[bce]	16(4/25)[e]	26.4(14/53)[b]	0.001
$CLBR^{hypo}$	51.2(44/86)[c]	77.3(34/44)[bce]	34.4(11/32)[e]	42.6(29/68)[b]	0.001

Data were presented as median (25th-75th percentile) for non-normality distribution variables. Gn: gonadotropin; LH: luteinizing hormone; E2: estradiol; P: progesterone; HCG: human chorionic gonadotropin IR: implantation rate; BPR: biochemical pregnancy rate; EPR: ectopic pregnancy rate; CPR: clinical pregnancy rate; MR: miscarriage rate; LBR: live birth rate CLBR: cumulative live birth rate, was calculated basing on current finished embryo transfer cycles. $CLBR^{hypo}$ was calculated by hypothesizing that all surplus embryos were transferred and live birth was achieved. [a]ultra-long vs short; [b]long vs short; [c]ultra-long vs long; [d]antagonist vs short; [e]long vs antagonist; [f]ultra-long vs antagonist [g]Fisher's exact test.

Supplementary Table VII Multivariate logistic regression of clinical pregnancy among women≥35 years in fresh ET cycles

	B	*SE*	*Wald*	*P*	*OR*	*95%CI*
Age	-0.240	0.091	6.932	0.008	0.787	0.658-0.941
BMI	0.044	0.075	0.350	0.554	1.045	0.903-1.210
Duration of infertility	-0.029	0.088	0.109	0.741	0.971	0.817-1.154
Primary infertility	control					
Secondary infertility	-0.930	0.817	1.297	0.255	0.394	0.080-1.955
AFC	-0.029	0.056	0.266	0.606	0.971	0.870-1.085
AMH	0.044	0.157	0.077	0.782	1.045	0.767-1.422
Basal FSH	0.171	0.090	3.591	0.058	1.186	0.994-1.415
initial uterine diameter	0.010	0.197	0.002	0.962	1.010	0.686-1.486
None dysmenorrhea	control					
Mild dysmenorrhea	-1.058	0.708	2.234	0.135	0.347	0.087-1.390
Moderate dysmenorrhea	-0.100	0.636	2.469	0.116	0.368	0.106-1.280
Severe dysmenorrhea	-1.079	0.644	2.805	0.094	0.340	0.096-1.202
COSGroup(short vs ultra-long)	0.045	0.838	0.003	0.957	1.046	0.202-5.402
COSGroup(short vs long)	-0.297	0.941	0.100	0.752	0.743	0.118-4.693
COSGroup(short vs antagonist)	0.288	1.052	0.075	0.785	1.333	0.17-10.480
COSGroup(antagonist vs ultra-long)	1.262	0.675	3.491	0.062	3.532	0.94-13.271
COSGroup(antagonist vs long)	1.044	0.723	2.083	0.149	2.841	0.69-11.726
COSGroup(long vs ultra-long)	1.070	0.612	3.053	0.081	2.915	0.878-9.683

BMI: body mass index; AFC: antral follicle count; FSH: follicle stimulating hormone; AMH: anti-müllerian hormone; COS: controlled ovarian stimulation;

Supplementary Table VIII Multivariate logistic regression of clinical pregnancy among women ≥35 years in FET cycles

	B	*SE*	*Wald*	*P*	*OR*	*95%CI*
Age	0.015	0.072	0.044	0.834	1.015	0.882-1.168
BMI	-0.115	0.068	2.854	0.091	0.892	0.781-1.019
Duration of infertility	0.021	0.072	0.082	0.774	1.021	0.887-1.174
Primary infertility	control					
Secondary infertility	-1.610	0.632	6.490	0.011	0.200	0.058-0.690
AFC	-0.101	0.049	4.341	0.037	0.903	0.821-0.994
AMH	0.099	0.110	0.811	0.368	1.104	0.890-1.371
Basal FSH	-0.069	0.094	0.543	0.461	0.933	0.777-1.121
initial uterine diameter	-0.192	0.160	1.434	0.231	0.825	0.602-1.130
None dysmenorrhea	control					
Mild dysmenorrhea	-0.820	0.622	1.739	0.187	0.441	0.130-1.490
Moderate dysmenorrhea	-0.330	0.556	0.353	0.552	0.719	0.242-2.136
Severe dysmenorrhea	-0.823	0.611	1.813	0.178	0.439	0.132-1.455
No GnRHa pretreatment	control					
GnRHa pretreatment	-0.365	0.395	0.856	0.355	0.694	0.320-1.505

BMI: body mass index; AFC: antral follicle count; FSH: follicle stimulating hormone; AMH: anti-müllerian hormone; GnRHa: gonadotropin agonist
